# First-reported pediatric cases of American ginseng anaphylaxis and allergy

**DOI:** 10.1186/s13223-018-0304-3

**Published:** 2018-11-03

**Authors:** Stephanie C. Erdle, Edmond S. Chan, Hyungjun Yang, Bruce A. Vallance, Christopher Mill, Tiffany Wong

**Affiliations:** 1 Division of Allergy & Immunology, Department of Pediatrics, University of British Columbia, British Columbia Children’s Hospital, 4480 Oak St., Vancouver, BC V6H3N1 Canada; 2 Division of Gastroenterology, Department of Pediatrics, British Columbia Children’s Hospital, Vancouver, Canada

**Keywords:** Ginseng, Pediatrics, Allergy, Anaphylaxis, Allergic conjunctivitis

## Abstract

**Background:**

Ginseng is a perennial herb used in traditional Chinese medicine, which has become increasingly popular world-wide due to its proposed medicinal effects. There are two major species of ginseng, *Panax ginseng* (Korean or Asian ginseng), and *Panax quinquefolius* (American ginseng). Although cases of allergy due to Korean ginseng have been reported in adults, there are no reported cases of allergy to American ginseng, and no reported cases of ginseng allergy in pediatric patients.

**Case presentation:**

We present two unique cases of pediatric patients with suspected allergic reactions to American ginseng. The first patient is a 6-year-old girl who presented to the emergency department in anaphylaxis (urticaria and respiratory symptoms) minutes after inhaling powdered American ginseng. There was evidence of sensitization to American ginseng on skin prick testing (SPT) (13 × 12 mm wheal) and evidence of allergy to American ginseng on basophil activation testing, with a dose-dependent increase in expression of CD63 on basophils in response to American ginseng extract. The second patient is a 3-year-old boy who presented with recurrent allergic conjunctivitis upon exposure to aerosolized powdered ginseng, with evidence of sensitization to American ginseng on SPT (13 × 7 mm wheal), but with no evidence of IgE-mediated allergic reaction during oral challenge with American ginseng powder.

**Conclusions:**

These cases highlight two different allergic responses to American ginseng in pediatric patients. To our knowledge, these are the first reported cases of allergy to American ginseng, in addition to the first reported cases of allergy to ginseng in pediatric patients.

## Background

Ginseng is a perennial herb used in traditional Chinese medicine, which has become increasingly popular world-wide due to its proposed medicinal effects, including improved cerebral function, glycemic control, pain relief, stress reduction, and many others [1–3]. There are two major species of ginseng, *Panax ginseng* (Korean or Asian ginseng), and *Panax quinquefolius* (American ginseng) [1]. Approximately 40 active ingredients have been identified in ginseng, but the pharmacological properties are attributed to their triterpene glycosides, also known as saponins or ginsenosides [3]. American and Korean ginseng have distinctive distributions of ginsenosides, which are thought to account for their differing proposed medicinal effects [3]. Cases of anaphylaxis, occupational asthma and allergic rhinoconjunctivitis due to Korean ginseng have been reported in Korean adults [4–8]. In contrast, there are no reported cases of allergy against American ginseng, or reported cases of ginseng allergy in pediatric patients. We present two unique cases of pediatric patients presenting with suspected allergy to American ginseng.

## Case presentations

### Patient 1: Ginseng-induced anaphylaxis

A 6-year-old girl with a history of multiple IgE-mediated food allergies, atopic dermatitis, and a remote history of asthma presented to the emergency department with urticaria, coughing, and wheezing. Symptoms began minutes after entering a ginseng store that was selling powdered American ginseng products. She did not have any respiratory symptoms or ingest anything prior to entering the store. Parents suspected she inhaled some powdered ginseng. This was her first known exposure to ginseng. On physical examination, she was afebrile with normal blood pressure for age. Respiratory examination confirmed increased work of breathing and decreased air entry with wheezing bilaterally. On dermatologic examination, she had urticaria on her chest. She was treated in the emergency department with salbutamol, dexamethasone and diphenhydramine. Symptoms resolved shortly after treatment, and the patient was referred to our Allergy Clinic.

In the Allergy Clinic, parents provided a history of wheezing with viral infections between ages two and four, with intermittent inhaled corticosteroid and salbutamol use. There had been no exacerbation of respiratory symptoms in over a year. She had confirmed food allergies to peanuts, tree nuts and fish, and had outgrown egg and wheat allergies. She was found to be sensitized to tree pollen.

**Fig. 1:**
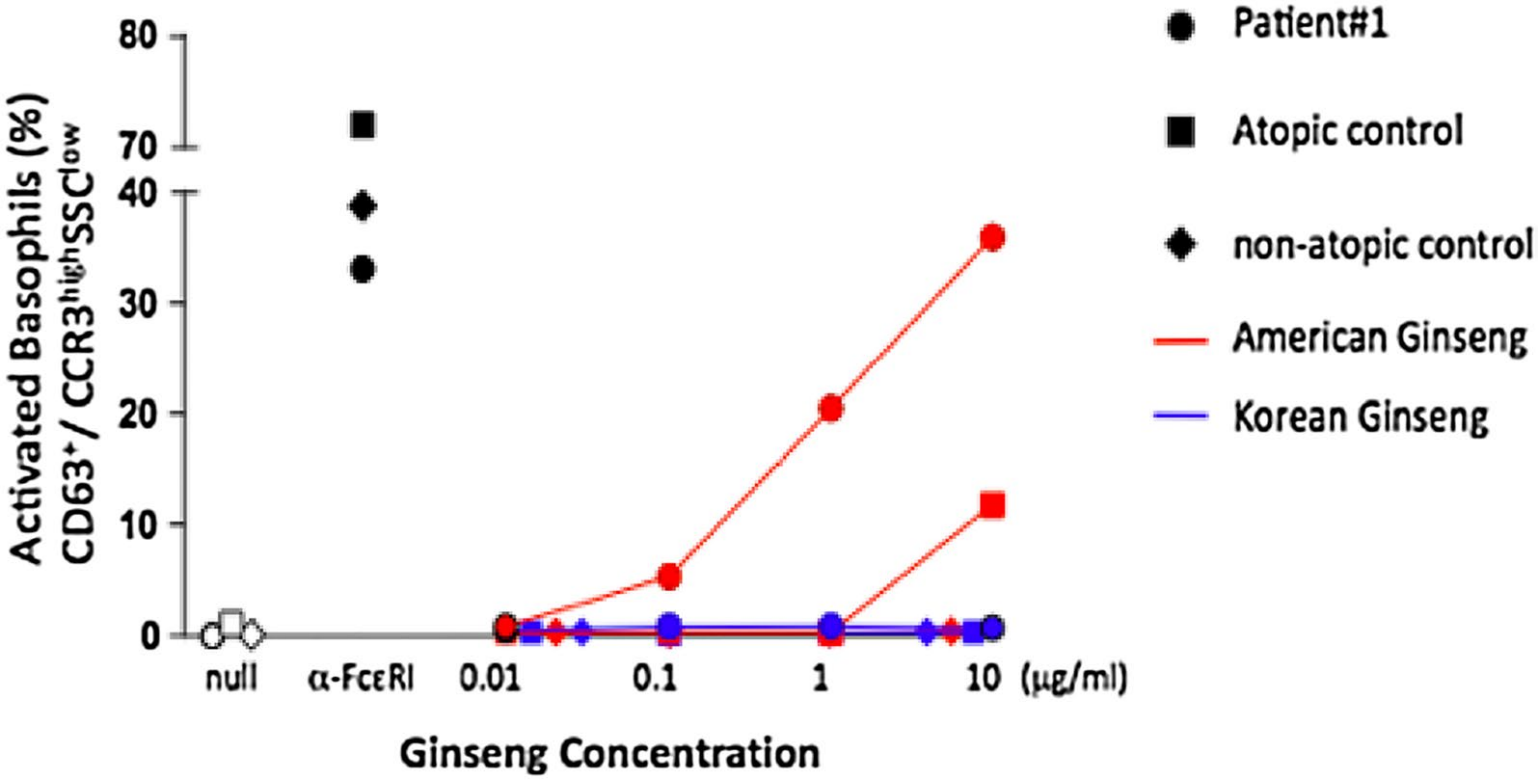
Expression of CD63 on basophils in Patient 1, an atopic control, and a non-atopic control, after incubation with American and Korean ginseng

Skin prick testing (SPT) with American ginseng powder dissolved in water was positive with a 13 × 12 mm wheal. Spirometry was normal (FEV1 107% predicted). The family declined an oral challenge to ginseng, given the severity of her initial reaction. Basophil activation test (BAT) showed a dose-dependent increase in expression of CD63 on basophils in response to American ginseng extract, but not Korean ginseng extract (Fig. 1). No changes were observed in a non-atopic control, and minimal changes were observed in an atopic control that was tested with American ginseng extract.

It was concluded that this patient had an anaphylactic reaction to American ginseng. She was advised to strictly avoid all ginseng products and carry an epinephrine autoinjector at all times.

### Patient 2: Ginseng-induced allergic conjunctivitis

A 3-year-old boy with asthma and atopic dermatitis was referred to our Allergy clinic with a history of recurrent ocular pruritus, tearing, and conjunctivitis. There were no associated nasal or respiratory symptoms. His ocular symptoms consistently occurred minutes after entering his grandparents’ herbal product store, on days when American ginseng was being ground. Symptoms typically resolved within 24 h of leaving the store, and did not occur when other herbs were being ground. There were no other identifiable triggers, and no seasonality to his symptoms. He was regularly ingesting soup boiled with small amounts of ginseng root without adverse reaction.

SPT with American ginseng powder dissolved in water was positive with a 13 × 7 mm wheal. SPT to common environmental aeroallergens was positive for dust mite. The family declined BAT due to needle phobia. An oral challenge was performed using American ginseng powder. Parents were asked to bring in 50 g of powdered ginseng [5], however, only brought in 3 g as they felt this quantity was too large. The powder was mixed with water, and ingested in increasing quantities. Parents stopped the challenge at 2 g, stating this was the maximum they would ever use in soup. He was monitored for 1 h following the challenge, and did not develop any signs of IgE-mediated allergy. The family was advised that it was likely safe for him to continue ingesting small amounts of ginseng in soup, but to avoid the grandparents’ store as much as possible, particularly on days when ginseng was being ground. He was given a prescription for olopatadine 0.1% eye drops for ocular symptoms as needed.

## Discussion and conclusion

Patient 1 presented in anaphylaxis, with physician-confirmed respiratory symptoms and urticaria. There was no recent history of viral infection or poor asthma control, and spirometry was normal, indicating that this presentation was unlikely to be an asthma exacerbation. There was evidence of sensitization to ginseng on SPT, and allergy on BAT. This is the first reported case of anaphylaxis to American ginseng. In addition, ginseng anaphylaxis has never been reported in a pediatric patient.

Patient 2 represents a unique case of allergic conjunctivitis on contact with aerosolized American ginseng powder. Interestingly, although his SPT was positive, suggesting an IgE-mediated response, he was able to tolerate the ingestion of small amounts of ginseng without the development of allergic symptoms. Kim et al. reported a similar entity in an adult patient who experienced recurrent rhinitis upon exposure to aerosolized ginseng with evidence of sensitization on SPT, but who was able to ingest ginseng without adverse reaction [4]. The pathophysiology behind this phenomenon is unclear, but may be related to the dilution effect of the protein and the very small amounts subsequently ingested.

Serum-specific IgE against American ginseng was not available at our center, but would have likely been more susceptible to false negatives than SPT with actual American ginseng.

These cases highlight two different allergic responses to American ginseng in pediatric patients; one with anaphylaxis, and the other with allergic conjunctivitis. As ginseng use and exposure is becoming increasingly prevalent world-wide, it is important to consider ginseng as a cause of anaphylaxis or allergic presentation in children. Further studies investigating the mechanism underlying the varied allergic presentations in response to ginseng would be valuable. In addition, the establishment of a standardized workup for the diagnosis of ginseng allergy would be beneficial.

## Abbreviations

BAT: basophil activation test
CD63: cluster of differentiation 63
FEV1: forced expiratory volume
IgE: immunoglobulin E
SPT: skin prick testing
